# Dose–response relationships between temporal variables of blood flow restriction exercise and physiological adaptations: a systematic review and meta-analysis

**DOI:** 10.3389/fphys.2026.1947412

**Published:** 2026-10-01

**Authors:** Yuda Li, Yi Wang, Sijie Wang, Tong Zhou

**Affiliations:** 1 School of Physical Education and Health Sciences, University of Lille, Lille, France; 2 Department of Anesthesiology, Jiangxi Cancer Hospital, Jiangxi, China; 3 Department of Physical Education, Northeastern University, Shenyang, China; 4 School of Strength and Conditioning, Beijing Sport University, Beijing, China

**Keywords:** blood flow restriction, exercise tolerance, hypoxic stimulus, physiological adaptations, temporal variables

## Abstract

**Background:**

To evaluate dose–response relationships between temporal variables of blood flow restriction (BFR) exercise as a physiological stimulus and changes in physiological adaptations.

**Methods:**

Following PRISMA, Web of Science, PubMed, SPORTDiscus and Scopus were searched (inception–3 May 2026) for RCTs in athletes or physically active healthy adolescents and young adults comparing BFR interventions with conventional exercise protocols. Risk of bias was assessed with RoB 2 and certainty with GRADE. In total, 15 articles (16 trials; 50 effect sizes) were included. Standardised mean differences (SMDs) were computed, with effect directions harmonised for context-dependent outcomes (HR, RER, RPE) so that positive values consistently reflect physiological improvements. These SMDs were pooled using a three-level random-effects model with robust variance estimation (RVE; clustered by study). A DerSimonian-Laird random-effects model with inverse-variance weighting was used for consistency checks.

**Results:**

Overall, BFR exercise elicited significant physiological adaptations (SMD = 0.249, 95% CI: 0.095–0.405), with findings remaining consistent in the DerSimonian-Laird model (overall SMD = 0.231, 95% CI: 0.111–0.349). By intensity, effects were: high-intensity BFR group (SMD = 0.208, 95% CI: 0.163–0.252), moderate-intensity group (SMD = 0.315, 95% CI: −0.465–1.095) and low-intensity group (SMD = 0.293, 95% CI: 0.057–0.529). Meta-regression showed positive associations of effect size with BFR duration (β = 0.0136, *p* = 0.0011) and stimulus duration (β = 0.0104, *p* = 0.0347). However, after adjusting for the period, BFR duration was not significant (*p* = 0.0972) due to collinearity; BFR pressure was not a significant moderator. Regarding specific physiological responses, only VO_2_max showed robust statistical significance (SMD = 0.386, 95% CI: 0.055–0.717).

**Conclusion:**

The most consistent evidence for BFR-induced improvements relates specifically to VO_2_max, whereas effects on other physiological outcomes remain tentative. In the initial RVE, among the specific physiological outcomes, VO_2_max, MPO, and RER reached significance, whereas HR and MAP were borderline; upon DL re-verification, only VO_2_max retained significance. Low-intensity BFR showed clearer advantages over conventional low-intensity exercise, whereas benefits were smaller versus conventional high-intensity protocols. Both BFR duration and stimulus duration showed positive dose–response relationships with effect size. However, multivariable models revealed that the apparent effect of BFR duration was confounded by the total intervention period. In addition, no other variables exhibited significant moderating effects.

## 1 Introduction

Blood flow restriction (BFR) applies external pressure to the proximal portion of a limb to partially reduce arterial inflow and markedly restrict venous return. By increasing metabolic stress, localized hypoxia, and neuromuscular recruitment, this ischemic model enables low-load exercise to evoke responses that resemble those typically induced by high-intensity stimuli, thereby promoting comparable physiological adaptations under reduced mechanical loading (Li et al., 2024). For instance, a recent randomized controlled trial demonstrated that adding BFR to a low-load multi-component program improves muscle thickness and functional outcomes versus the identical program without BFR (Ahmad et al., 2026). Proposed mechanisms include greater local hypoxia and metabolite accumulation, enhanced feedback from group III/IV afferents, earlier recruitment of high-threshold motor units, and shifts in intracellular signaling and endocrine/paracrine environments toward an anabolic profile, ultimately improving physiological capacities. Although BFR has been widely investigated in clinical and rehabilitative settings (e.g., sarcopenia, neurological disorders, and postoperative recovery) (Feng et al., 2025; Jønsson et al., 2024; Patterson et al., 2019), its application in sports science has increasingly shifted toward healthy, physically active individuals and athletes. In these populations, BFR paradigms have expanded beyond traditional low-load resistance exercise to walking, treadmill running, cycling, aerobic interval training, and endurance modalities. Accordingly, research has moved beyond whether BFR merely preserves muscle mass to whether it can add specific physiological benefits without increasing overall mechanical load. While recent BFR meta-analyses have predominantly synthesized evidence based on external loading intensity or applied occlusion pressure (Lixandrão et al., 2018), the hypoxic and metabolic stress induced by BFR is inherently time-dependent. Consequently, “temporal variables”—operationally defined in this review as the time-related dosing parameters of a BFR protocol, including the BFR duration per session, stimulus duration, training frequency, and total intervention period—have emerged as critical, yet underexplored, modulators of physiological adaptation. Because prolonged ischemic exposure may optimize metabolic accumulation while excessive restriction limits full reperfusion, understanding this temporal dimension is essential for safe and effective prescription. However, whether distinct dose–response relationships exist between these temporal variables and heterogeneous outcomes—such as maximal oxygen uptake (VO_2_max), power output, and neuromuscular adaptations—remains insufficiently synthesized. To address this specific gap and advance the current literature, we conducted a systematic review and meta-analysis strictly focusing on athletes and active healthy adolescents and young adults with prior exercise experience. By isolating time-related variables as potential moderators, our goal was to determine dose–response relationships and provide precise, evidence-based insights into optimizing temporal protocols for physiological adaptations. Specifically, we hypothesized that longer BFR duration and stimulus duration would demonstrate positive dose-response associations with the magnitude of physiological adaptations. Furthermore, to ensure methodological transparency, univariate meta-regressions for continuous temporal variables and intensity-based subgroup analyses were pre-registered *a priori* in PROSPERO, whereas the subsequent tertile-based categorizations were conducted *post hoc* for exploratory interpretation.

## 2 Materials and methods

This systematic review and meta-analysis followed the Preferred Reporting Items for Systematic Reviews and Meta-Analyses (PRISMA) 2020 guidelines (Page et al., 2021) and was prospectively registered in PROSPERO (CRD420261291228).

### 2.1 Literature search strategy

Two independent reviewers systematically searched PubMed, Scopus, Web of Science, and SPORTDiscus from database inception to May 3, 2026. The search strategy was developed through expert consultation and comprehensively expanded to capture a broad range of physiological and performance adaptations. The search string combined intervention-related keywords and Medical Subject Headings (MeSH) (e.g., “Blood flow restriction”, “KAATSU”, “vascular occlusion”) with an extensive list of outcome-specific terms (e.g., “VO_2_max”, “muscle strength”, “heart rate”, “exercise capacity”, “athletic performance”). To maximize search sensitivity and prevent the inadvertent exclusion of relevant trials, population-specific terms (e.g., “athletes”) were deliberately omitted from the search string, leaving population selection strictly to the manual screening phase. Furthermore, the search strategy was iteratively refined and validated against a pre-identified set of known eligible sentinel studies to ensure exhaustive retrieval (Bramer et al., 2018). Where database functionality permitted, standard search filters for human subjects and randomized controlled trials were systematically applied. The restriction to English-language publications was necessitated by resource limitations regarding translation; we acknowledge this as a potential source of language bias. Furthermore, reference lists of included studies and relevant prior reviews were hand-searched to minimize the risk of missing eligible records. The complete, database-specific search strategies, including exact syntax and MeSH terms tailored for each platform, are detailed in **Supplementary Table 1**.

### 2.2 Study selection and eligibility criteria

One reviewer screened titles and abstracts, and two independent reviewers assessed full texts. Disagreements were resolved by discussion or, when necessary, by consultation with a third reviewer. References were managed in EndNote. Inter-rater agreement during screening was high (*k* = 0.91, *p* < 0.001). Eligibility criteria were structured using the PICOS framework (Methley et al., 2014): (1) Population: athletes or physically active healthy adolescents and young adults; (2) Intervention: BFR interventions; (3) Comparator: conventional exercise of the same or different intensity; (4) Outcomes: physiological adaptations and specific physical performance metrics; (5) Study design: randomized controlled trials (RCTs). Studies were excluded if they were: (1) Non-RCT designs; (2) Conducted in ineligible populations (e.g., older adults or individuals requiring sports injury/rehabilitation); or (3) Based on non-conforming experimental designs. Because this review focused on long-term adaptive effects, we excluded studies that used BFR only as an acute, single-bout intervention without a periodized intervention regimen.

### 2.3 Data extraction

Two reviewers independently extracted data using a standardized form. Extracted information included: (1) Study design characteristics; (2) Participant baseline characteristics (age and sex); (3) BFR variables (exercise intensity, stimulus duration, frequency, intervention period, BFR duration, and BFR pressure); and (4) Outcomes related to physiological adaptations, including maximal oxygen uptake (VO_2_max), maximal aerobic power (MAP), peak power output (PPO), mean power output (MPO), heart rate (HR), respiratory exchange ratio (RER), countermovement jump (CMJ), one-repetition maximum (1RM), and rating of perceived exertion (RPE). When a study reported multiple eligible outcomes, each was treated as an independent effect size. Data presented only graphically were digitized using Web Plot Digitizer (v4.5). To ensure methodological transparency and standardize the dose–response analyses, explicit operational definitions and extraction criteria for all BFR variables and physiological outcomes are detailed in **Table 1**.

**Table 1:** Operational definitions and extraction criteria for BFR variables and physiological adaptations.

Category	Variable	Unit/scale	Operational definition/extraction criteria
BFR Variables	BFR intensity	Categorical (Low, Moderate, High)	The magnitude of external load. Categorized based on standard guidelines (e.g., ACSM) or as explicitly stated in the original studies: Resistance (%1RM): Low (≤40%); Moderate (41–69%); High (≥70%). Aerobic (VO_2_max/HRR): Low (<40%); Moderate (40–59%); High (≥60%).
	BFR pressure (Occlusion pressure)	mmHg or % AOP	The mechanical pressure exerted by the tourniquet.
	Stimulus duration	Minutes (min)	The total duration of the training session. This metric encapsulates the entire exercise bout from start to finish, including all active exercise sets, inter-set rest intervals, and warm-up/cool-down periods.
	BFR duration	Minutes (min)	The continuous, uninterrupted time the pneumatic cuff remains inflated per session. Crucially, this includes all inter-set rest/reperfusion intervals if the cuff is not deflated.
	Frequency	Sessions/week	The total number of completed BFR training sessions per week. Evaluated as a temporal moderator.
	Period	Weeks (wk)	The macroscopic temporal span of the entire BFR training protocol, from baseline to post-intervention.
Physiological outcomes	Maximal Oxygen Uptake (VO2max)	mL/kg/min or L/min	The maximum rate of oxygen consumption during exhaustive exercise.
	Maximal Power Output (MPO)	Watts (W)	The highest mechanical power generated during maximal effort. Reflects the synergistic adaptation of aerobic power and neuromuscular capacity.
	Respiratory Exchange Ratio (RER)	Dimensionless (VCO_2_/VO_2_)	The ratio of carbon dioxide production to oxygen consumption. Effect sizes were harmonized so that positive SMDs reflect physiological improvements.
	Heart Rate (HR)	Beats per minute (bpm)	Cardiac cycles per minute. Effect sizes were harmonized so that positive SMDs reflect physiological adaptations (e.g., decreased submaximal HR).
	Mean Arterial Pressure (MAP)	mmHg	The average arterial pressure representing organ perfusion. Serves as a marker for vascular adaptation and hemodynamic remodeling.
	Rating of Perceived Exertion (RPE)	Borg Scale (e.g., 6–20)	A psychophysiological proxy for internal load and perceptual adaptation. Effect sizes were harmonized so that positive SMDs reflect a decrease in perceived exertion at identical workloads.
	One-Repetition Maximum (1RM)	Kilograms (kg) or Pounds (lbs)	The maximal external load that can be lifted through a full range of motion for one complete repetition. Serves as the gold-standard indicator for dynamic maximal muscle strength.
	Countermovement Jump (CMJ)	Centimeters (cm) or Meters (m)	A maximal vertical jump initiated with a downward eccentric countermovement. Evaluates lower-limb explosive power, stretch-shortening cycle (SSC) efficiency, and reactive neuromuscular performance.

### 2.4 Risk of bias assessment

Risk of bias was evaluated using the Cochrane Risk of Bias tool (RoB 2) (Sterne et al., 2019), which covers five domains: randomization process, deviations from intended interventions, missing outcome data, measurement of the outcome, and selection of the reported result. Certainty of evidence was graded with the Grading of Recommendations Assessment, Development and Evaluation (GRADE) system (Andrews et al., 2013). Evidence was downgraded (high, moderate, low, or very low) for risk of bias, inconsistency, indirectness, imprecision, or publication bias. Assessments were performed independently by two reviewers, with disagreements resolved by a third methodological expert according to the Cochrane Handbook for Systematic Reviews of Interventions (version 6.5). Consistent with Cochrane guidance (Page et al., 2024), publication bias was evaluated separately by BFR intensity using funnel plots. Because the number of independent study clusters within each intensity stratum (n = 7, 6, and 3) fell below the recommended threshold of 10, and because individual studies contributed multiple dependent effect sizes, we did not perform conventional Egger’s quantitative regression or trim-and-fill analyses. Conducting such tests under these conditions violates the statistical assumption of independence, artificially inflates degrees of freedom, and increases the risk of Type I errors. Consequently, publication bias was strictly assessed qualitatively through the visual inspection of funnel plot symmetry.

### 2.5 Statistical analysis

Statistical analyses were performed in R (version 4.4.1) using the metafor package (version 4.8-0). Effect sizes were expressed as standardized mean differences (SMDs; Hedges’ *g*) and weighted by the inverse of their sampling variances. Each eligible outcome generated a separate effect size. To compute Hedges’ g, mean change scores (post-intervention minus pre-intervention) and their corresponding standard deviations (SDs) were exclusively utilized to adjust for potential baseline imbalances. For multi-arm trials sharing a single control group, we circumvented unit-of-analysis errors by evenly dividing the sample size of the shared control group across the active intervention arms (Higgins et al., 2024). Additionally, in accordance with standard meta-analytic guidelines (Higgins et al., 2024), prior to pooling, the mathematical signs of effect sizes for HR, RER, and RPE were inverted so that a positive SMD consistently represented a favorable physiological adaptation. Specifically, for submaximal assessments where a physiological decrease indicates improvement (e.g., lower submaximal HR, RER, or RPE at identical absolute workloads), signs were inverted to harmonize effect directions. To ensure absolute analytic transparency, this procedure was applied to the data extracted from specific relevant trials (e.g., Amani-Shalamzari et al., 2020; Chen et al., 2022; Giovanna et al., 2022; Thompson et al., 2024). Because these specific outcomes are inherently context-dependent, this harmonization was necessary to compute a macroscopic dose-response model. However, we acknowledge that unifying these directions may aggregate distinct physiological systems (e.g., cardiovascular stress versus substrate metabolism), thereby limiting specific mechanistic interpretations. To accommodate dependent effect sizes (i.e., multiple effects from the same study), we used a three-level random-effects model (rma.mv; effect sizes nested within studies) to estimate overall and moderating effects. Robust variance estimation (RVE) was applied with the study as the clustering unit (cluster-robust sandwich estimator), and inference relied on *t*- and *F*-tests with small-sample degrees-of-freedom correction (CR1), yielding robust standard errors, 95% confidence intervals, and *p*-values (Deeks et al., 2024; Sterne et al., 2011). Univariate meta-regressions for continuous temporal variables and intensity-based subgroup analyses were specified *a priori* in our PROSPERO protocol. However, to facilitate interpretation, continuous duration variables were subsequently categorized into data-driven tertiles for additional subgroup comparisons. While such quantile-based dose stratification improves descriptive clarity and visualizes trends without subjective cut-points, we acknowledge this *post hoc* categorization inherently reduces statistical power, eliminates within-category distance information, and introduces cut-point dependency (Altman and Royston, 2006; Greenland, 1995; Royston et al., 2006). To ensure the stability of the variance estimates, a minimum-cluster threshold was explicitly defined: When the number of independent studies was insufficient for RVE (i.e., fewer than five independent clusters), we reported point estimates descriptively. For comparability with prior work and to evaluate the consistency of point estimates, we conducted a supplementary DerSimonian-Laird random-effects analysis with inverse-variance weighting. In the primary analysis, heterogeneity was evaluated via residual heterogeneity and variance components; the *Q* statistic was additionally reported for the DerSimonian-Laird models. Publication bias was examined strictly through the qualitative visual inspection of funnel plots, stratified by exercise intensity to avoid misclassifying true between-stratum heterogeneity as small-study effects. Quantitative asymmetry tests (e.g., Egger’s test) were precluded to avoid unit-of-analysis errors and Type I error inflation, given the dependent effect sizes and the limited number of independent study clusters (n < 10) per stratum. Sensitivity analyses included leave-one-study-out procedures and comparisons across key model specifications.

## 3 Results

### 3.1 Literature search results

The database search identified 1,829 records. After duplicate removal, 1,647 records remained for title/abstract screening. After excluding clearly irrelevant reports and those not meeting inclusion criteria, 149 articles were retained for full-text review. Following full-text assessment, 44 articles proceeded to the final eligibility check. At this stage, 29 studies were excluded due to non-conforming methods (*n* = 5), ineligible participants (*n* = 17), or non-qualifying outcomes (*n* = 7). In total, 15 articles (16 trials) were included, yielding 50 effect sizes for quantitative synthesis. The selection process is shown in the PRISMA flow diagram (**Figure 1**).

**Figure 1:**
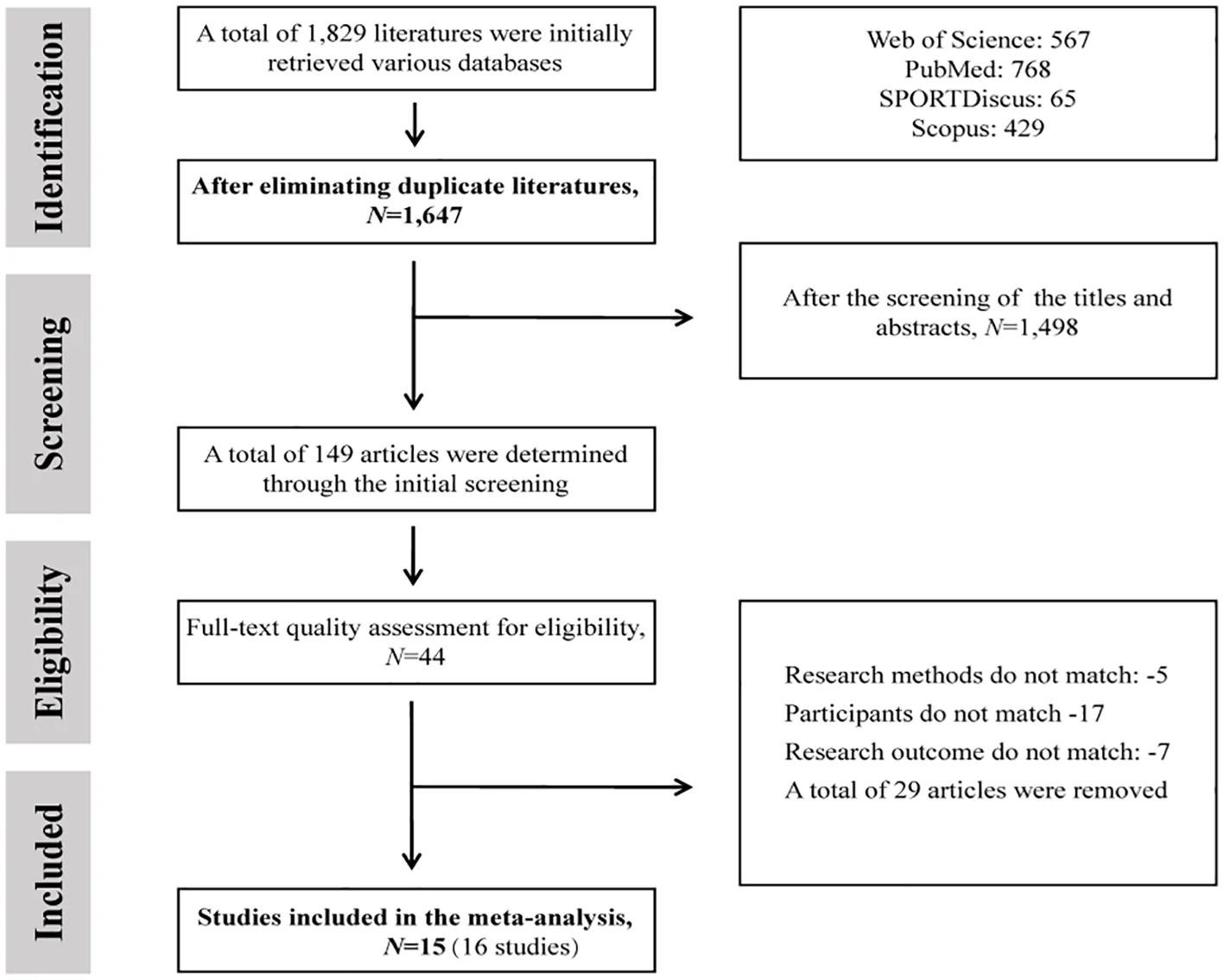
PRISMA flow diagram.

### 3.2 Characteristics of included studies

Fifteen articles (16 studies) reporting nine outcome measures were included, contributing 50 effect sizes (**Table 2**). Participants were athletes or physically active healthy adolescents and young adults, with a mean age of 23.87 years [range: 15.9 years (Kakhak et al., 2022) to 30.2 years (Bradley et al., 2023; Beak et al., 2022)]. Samples were predominantly male or mixed-sex; no study included only female participants. By intervention intensity, six studies used high-intensity BFR, three used moderate-intensity BFR, and seven used low-intensity BFR. BFR pressure was reported as a percentage of arterial occlusion pressure (%AOP) in 5 studies (mean: 86.00%) and as millimeters of mercury (mmHg) in 11 studies (mean: 162.27 mmHg). Additionally, further methodological details for the remaining included studies can be found in their respective original reports (Corvino et al., 2014; Taylor et al., 2016; Amani et al., 2018; Torpel et al., 2018; Mitchell et al., 2019; Daryani and Borkar, 2020; Held et al., 2020; Chen et al., 2025). Stimulus duration ranged from 15 min (Beak et al., 2022) to 69 min (Kakhak et al., 2022) (mean: 29.11 min), and BFR duration per session ranged from 4.8 min (Giovanna et al., 2022) to 55 min (Kakhak et al., 2022) (mean: 17.39 min). Frequency averaged 2.79 sessions per week, ranging from 2 sessions per week to 5 sessions per week. Period ranged from 2 weeks to 8 weeks (mean: 4.54 weeks).

**Table 2:** Characteristics of included studies.

Study	Year	Sex	Age	Sample size	BFR intensity	BFR pressure	Stimulus duration	BFR duration	Frequency	Period	Comparator description	Outcome
Chen et al.	2025	Male	19.7	33	Moderate	190*	20 min	16 min	2 sess	6 wks	High-intensity interval training (HIIT)	A; B; D; I
Thompson et al. a	2024	Mix	26	28	High	100%	25 min	13 min	5 sess	4 wks	Interval treadmill walking (5 km/h, 5% grade)	A; B; C; G
Thompson et al. b	2024	Mix	26	28	Low	100%	25 min	13 min	2 sess	6 wks	Interval treadmill walking (5 km/h, 5% grade)	A; B; C; G
Bradley et al.	2023	Mix	30.2	20	Low	80%	21 min	11 min	2 sess	4 wks	High-intensity rowing and deadlifts	A; B; J
Chen et al.	2022	Male	21.6	20	Moderate	153.8*	19 min	19.5 min	3 sess	8 wks	Moderate-intensity (50% HRR) interval running	A; C; I
Kakhak et al.	2022	Male	15.9	19	Low	185*	69 min	55 min	3 sess	6 wks	Soccer-specific drills and small-sided games	D; J
Giovanna et al.	2022	Male	25.6	20	High	88.2*	44 min	4.8 min	3 sess	2 wks	Repeated sprint training (RST) cycling	A; B; C; G
Beak et al.	2022	Male	30.2	30	Low	200*	15 min	10 min	3 sess	8 wks	Low-intensity interval running (40% VO_2_max)	A; B
Amani-Shalamzari et al.	2020	Male	23.9	19	Moderate	175*	19.8 min	15 min	3.3 sess	3 wks	3v3 small-sided futsal games	A; I
Held et al.	2020	Mix	21.9	31	Low	75%	30 min	20 min	3 sess	5 wks	Low-intensity rowing	A; J
Daryani et al.	2020	Mix	21.5	30	Low	75%	20 min	14 min	2.5 sess	4 wks	Continuous treadmill running (40–50% HRR)	A
Mitchell et al.	2019	Male	23	21	High	120*	28 min	18 min	2 sess	4 wks	Sprint interval training (SIT) cycling	A; B; E; F
Amani et al.	2018	Male	23	12	High	110*	28 min	18 min	4 sess	2 wks	400-m interval running	A; B; C
Torpel et al.	2018	Male	23.4	24	High	273*	22 min	14 min	3 sess	4 wks	High-intensity interval training (HIIT) cycling	A; B; C; D; J
Taylor et al.	2016	Male	27	20	High	130*	28 min	11 min	2 sess	4 wks	Sprint interval training (SIT) cycling	A; B; E; F
Corvino et al.	2014	Mix	23	13	Low	160*	52 min	26 min	3 sess	4 wks	Low-intensity interval cycling (30% Peak Power)	B

1. “*” denotes mmHg; 2. “%” denotes AOP%; 3. “sess” denotes sessions/week; 4. “wks” denotes weeks; 5. “A” denotes VO_2_max; 6. “B” denotes Peak Power; 7. “C” denotes HR; 8. “D” denotes CMJ; 9. “E” denotes MAP; 10. “F” denotes MPO; 11. “G” denotes RER; 12. “I” denotes RPE; 13. “J” denotes 1RM.

### 3.3 Quality assessment of included studies

Most included studies were rated as having “some concerns” for the overall risk of bias, largely reflecting the practical challenges of blinding participants and personnel in exercise interventions, which primarily affected the domain of deviations from intended interventions (**Figure 2**). Additionally, several studies provided insufficient details regarding random sequence generation or allocation concealment (affecting the randomization process), and many lacked pre-registered analysis plans (affecting the selection of reported results). By contrast, the measurement of outcomes domain was unanimously rated as low risk (100%), as the physiological outcomes were predominantly assessed objectively. Missing outcome data were typically minimal or handled appropriately. Overall, methodological quality was considered acceptable, but the remaining uncertainties warrant cautious interpretation. The certainty of evidence for all pooled physiological outcomes was assessed using the GRADE framework. Although RCT evidence initially starts at high certainty, ratings were downgraded for risk of bias, inconsistency, indirectness, imprecision, or potential publication bias as needed. Crucially, the aforementioned “some concerns” ratings in the RoB-2 assessment were deemed serious enough to plausibly influence effect estimates, thereby prompting a direct one-level downgrade in the GRADE risk of bias domain. When other GRADE domains were also compromised, the certainty was further downgraded. Consequently, the overall certainty of evidence was most commonly judged as moderate, with some specific outcomes rated as low certainty.

**Figure 2:**
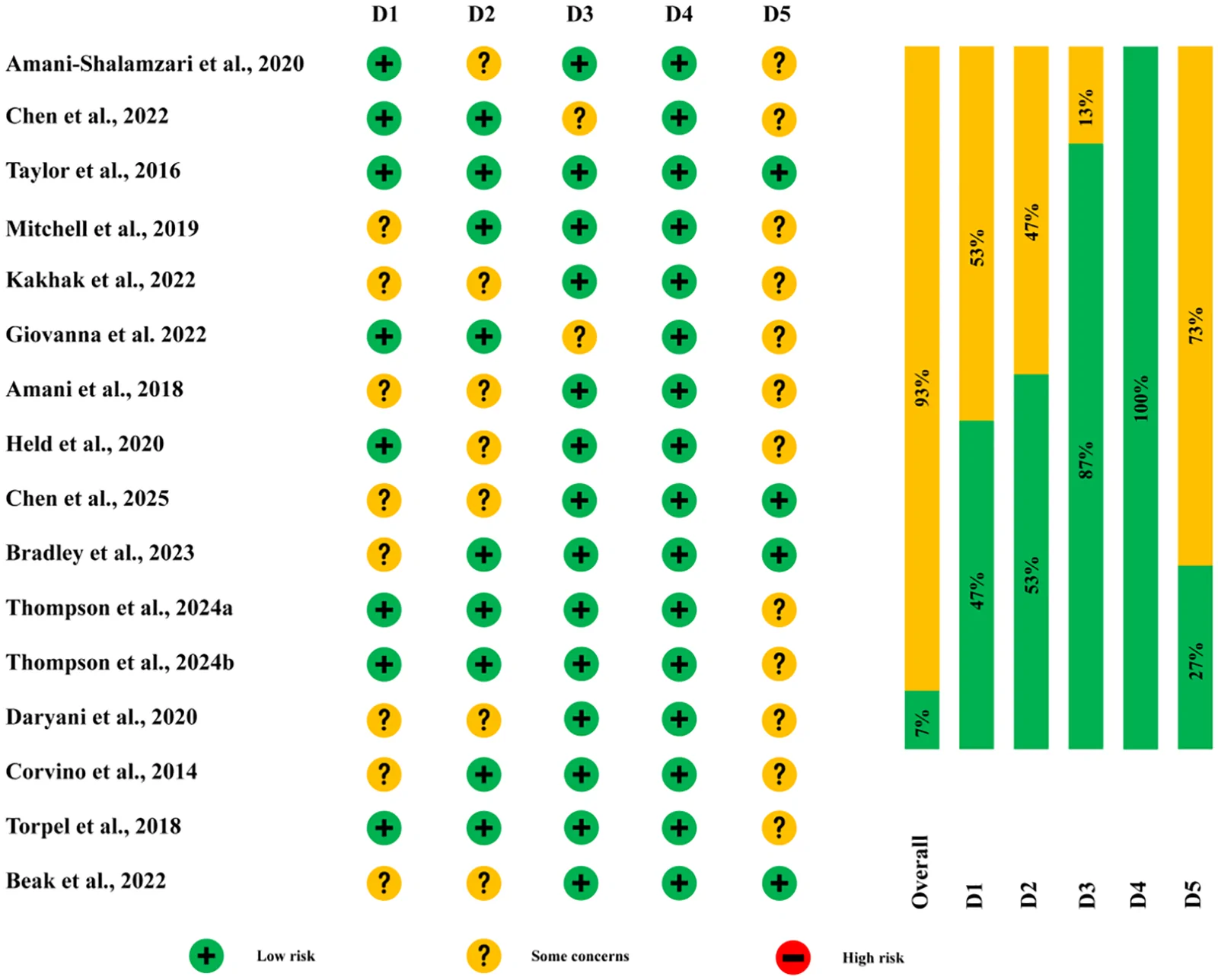
Quality assessment of included studies.

Notes: D1: Bias arising from the randomization process; D2: Bias due to deviations from the intended interventions; D3: Bias due to missing outcome data; D4: Bias in measurement of the outcome; D5: Bias in selection of the reported result.

### 3.4 Main results

#### 3.4.1 Effects of BFR at different intensities and meta-regression analysis of temporal variables

A three-level random-effects model (REML estimator) with cluster-robust variance estimation (RVE; CR1 type with small-sample degrees-of-freedom correction) was used for inference (*k* = 50 effect sizes, clusters = 16 studies) (**Figure 3A**). After including intensity as a moderator, residual heterogeneity was not significant (*Q*E(47) = 37.61, *p* = 0.834). The pooled estimate indicated a small-to-moderate positive overall effect (SMD = 0.249, 95% CI: 0.095–0.405, *p* = 0.0037). By intensity, the high-intensity BFR group showed a significant and precise effect (SMD = 0.208, 95% CI: 0.163–0.252, *p* < 0.0001), and the low-intensity BFR group also showed a significant positive effect (SMD = 0.293, 95% CI: 0.057–0.529, *p* = 0.019). The moderate-intensity BFR group had a positive but non-significant estimate with a wide confidence interval (SMD = 0.315, 95% CI: −0.465–1.095, *p* = 0.399), indicating an imprecise and likely underpowered estimate. To examine potential moderators, stimulus duration, BFR duration, BFR pressure, stimulus frequency, and total intervention period were entered into separate univariate meta-regression models (derived from 16 independent study clusters). BFR duration was positively associated with effect size (β = 0.0136, *SE* = 0.0033, 95% CI: 0.0065–0.0207, *p* = 0.0011), and stimulus duration also showed a positive association (β = 0.0104, *SE* = 0.0045, 95% CI: 0.0009–0.0200, *p* = 0.0347). Residual heterogeneity remained non-significant in both moderator models (*Q*E *p* > 0.93) (**Figure 4**). No other variables showed statistically significant moderating effects. To address potential collinearity between session-level durations and the total intervention period, multivariable meta-regression models were conducted. After adjusting for total intervention weeks, stimulus duration remained a significant positive moderator (β = 0.0122, 95% CI: 0.0009–0.0235, *p* = 0.0342). However, the univariate association for BFR duration was attenuated and lost statistical significance (β = 0.0126, 95% CI: −0.0023–0.0274, *p* = 0.0972). This indicates that the previously observed effect of BFR duration was primarily confounded by the total training period, and its univariate results should be interpreted with caution.

**Figure 3:**
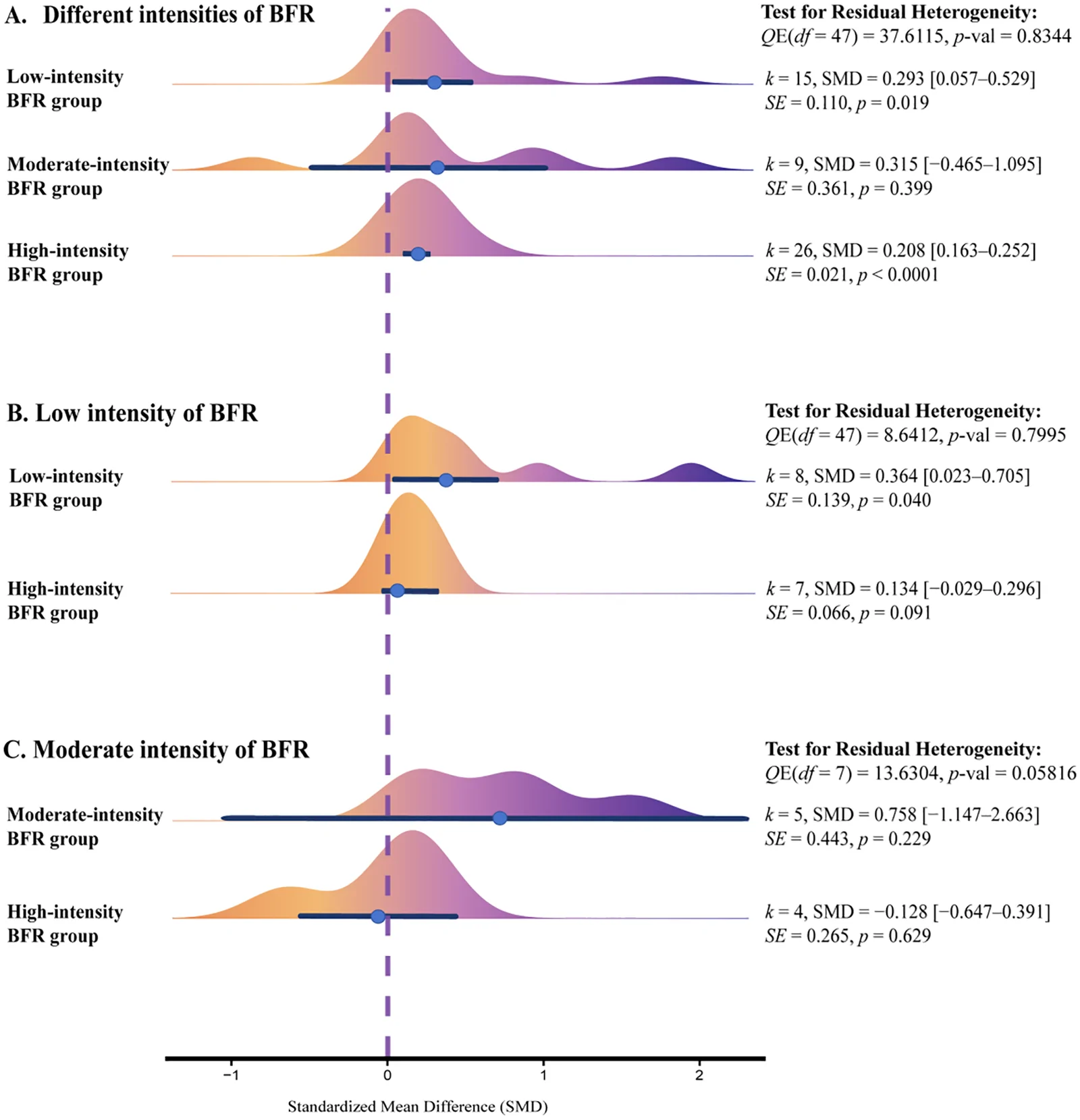
Forest plots of physiological adaptations stratified by BFR exercise intensity. **(A)** Different intensities of BFR; **(B)** Low intensity of BFR; **(C)** Moderate intensity of BFR.

**Figure 4:**
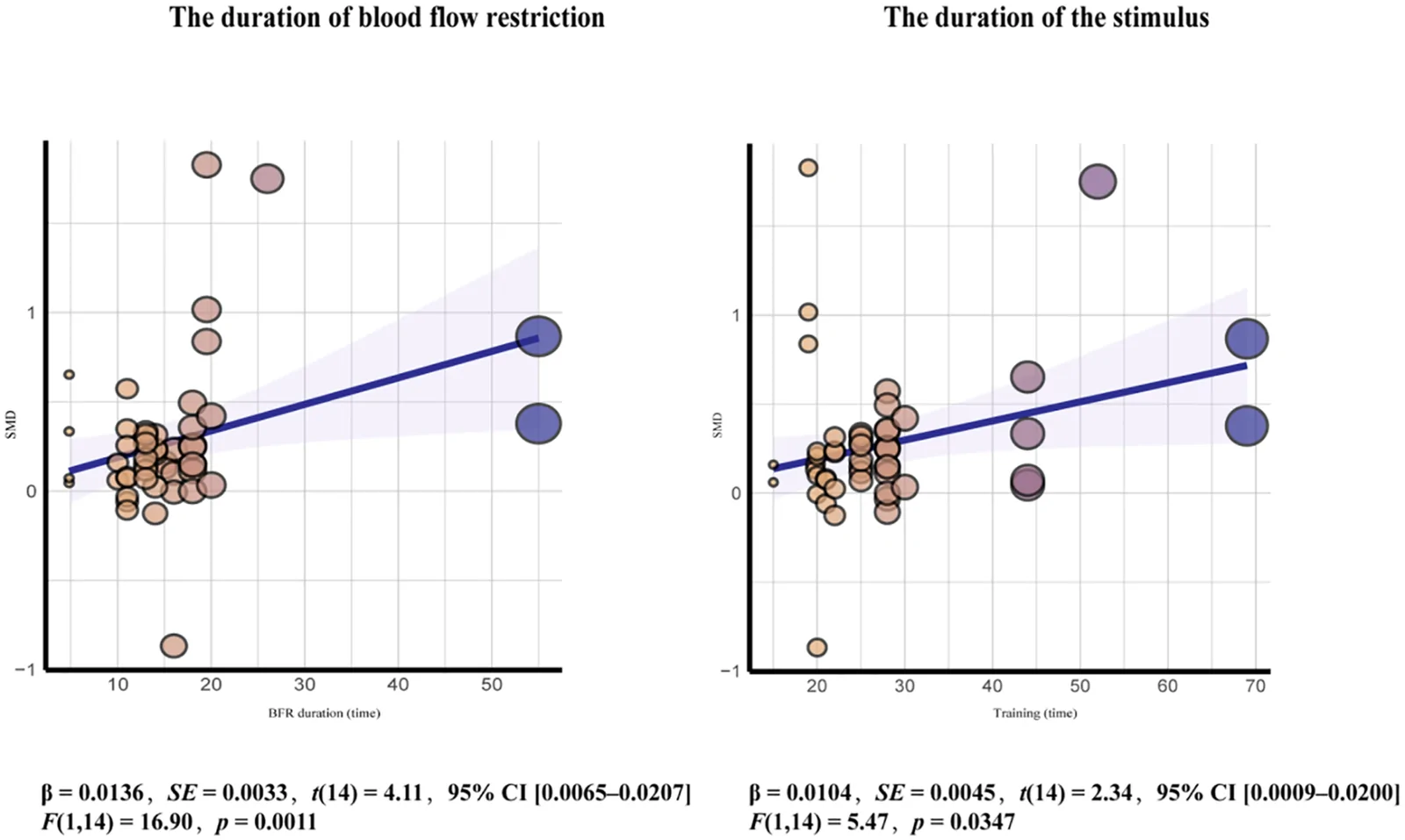
Univariate meta-regression analyses of temporal BFR variables on physiological adaptations.

#### 3.4.2 Comparative analysis of control groups of different intensities

When studies were stratified by comparator intensity, the high-intensity BFR subgroup was compared exclusively with conventional high-intensity exercise. Accordingly, its pooled estimate was unchanged. Low-intensity BFR significantly outperformed conventional low-intensity protocols (SMD = 0.364, 95% CI: 0.023–0.705, *p* = 0.040). Compared with conventional high-intensity exercise, low-intensity BFR showed a positive but non-significant trend (SMD = 0.134, 95% CI: −0.029–0.296, *p* = 0.091) (**Figure 3B**). For moderate-intensity BFR versus conventional high-intensity exercise, the limited number of independent studies/clusters precluded reliable RVE-based inference. A supplementary DerSimonian-Laird random-effects model also showed no significant effect (SMD = −0.128, 95% CI: −0.647–0.391, *p* = 0.629). Relative to conventional moderate-intensity exercise, the point estimate was positive but non-significant with a very wide confidence interval (SMD = 0.758, 95% CI: −1.147–2.663, *p* = 0.229), suggesting that non-significance likely reflects limited data and imprecision rather than a reversal of effect direction (**Figure 3C**). As noted above, the high-intensity BFR subgroup remained consistent with the primary intensity-stratified analysis (SMD = 0.208, 95% CI: 0.163–0.252, *p* < 0.0001).

#### 3.4.3 Comparative analysis of different BFR durations

BFR duration was categorized into three groups (short, moderate, long) using a tertile split for exploratory analyses (**Figure 5A**). The short time group (4.8–13 min; *n* = 6 clusters, *k* = 22 effect sizes) showed a statistically significant positive effect with relatively precise estimates (SMD = 0.185, 95% CI: 0.120–0.250). The moderate time group (14–18 min; *n* = 6 clusters, *k* = 20) demonstrated a smaller, non-significant effect (SMD = 0.093, 95% CI: −0.057–0.243). The long-time group (19.5–55 min; *n* = 4 clusters, *k* = 8) exhibited the largest and statistically significant effect (SMD = 0.719, 95% CI: 0.212–1.225). In strict accordance with methodological best practices, because the number of independent clusters per tertile was highly limited (*n* < 10), RVE-based p-values have been omitted to prevent unreliable Type I error inflation. Consequently, these specific 95% CIs and point estimates must be interpreted strictly descriptively. Overall, these patterns provide supportive exploratory evidence consistent with the primary RVE meta-regression, suggesting a positive association between longer BFR duration and greater effect magnitude.

**Figure 5:**
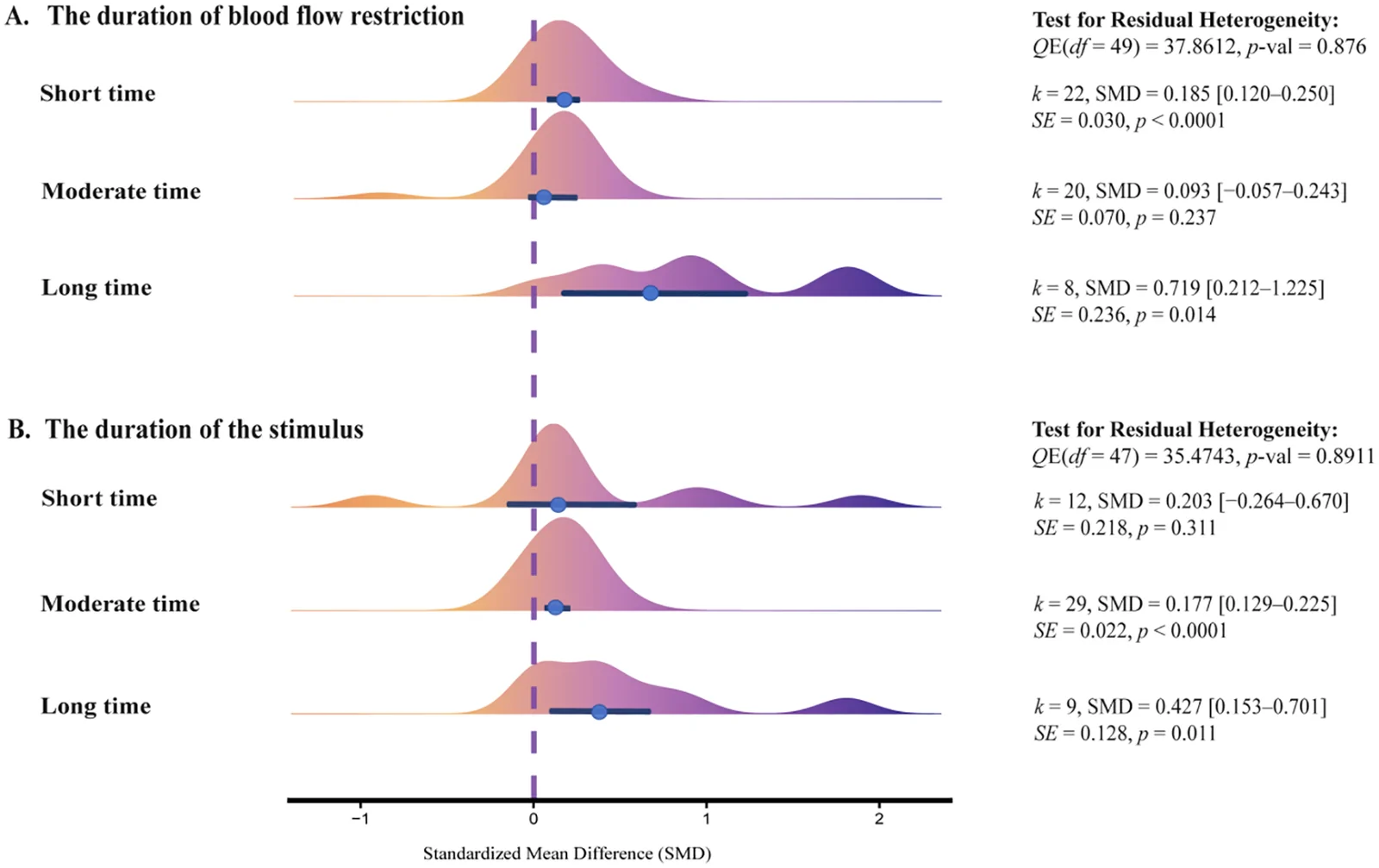
Exploratory subgroup analyses of BFR duration and stimulus duration categorized by tertiles. **(A)** The duration of blood flow restriction; **(B)** The duration of the stimulus.

#### 3.4.4 Comparative analysis of different stimulus durations

Stimulus duration was categorized into tertiles (short time: ≤ 20 min; moderate time: 21–29 min; long time: ≥ 30 min) for exploratory subgroup analyses (**Figure 5B**). Similar to the BFR duration subgroups, independent clusters per tertile were limited (*n* < 10), necessitating a strictly descriptive interpretation without formal hypothesis testing (p-values omitted). A descriptive gradient pattern was observed, similar to that for BFR duration. The long-time group (*n* = 4 clusters, *k* = 9 effect sizes) showed a significant positive effect (SMD = 0.427, 95% CI: 0.153–0.701), and the moderate time group (*n* = 7 clusters, *k* = 29) also showed a significant and more precisely estimated effect (SMD = 0.177, 95% CI: 0.129–0.225). The short time group (*n* = 5 clusters, *k* = 12) had a positive but non-significant estimate with substantial uncertainty (SMD = 0.203, 95% CI: −0.264–0.670), indicating limited evidence in this range. Overall, moderate and longtime groups may be associated with greater BFR effects; this analysis complements the significant association observed in the meta-regression for stimulus duration. Because tertile thresholds are data-driven and may introduce information loss and imbalance across groups, these findings are observational rather than prescriptive, and should be interpreted merely as supplementary to the model-based meta-regression. Consequently, they should not be directly applied as standardized training protocols in practice and warrant cautious interpretation.

#### 3.4.5 Comparative analysis of different physiological outcome measures

Outcome-stratified analyses showed no significant residual heterogeneity (*Q*E(41) = 32.71, *p* = 0.819). Preliminary results indicated that most outcomes had positive point estimates, although the strength of evidence varied. MPO showed a significant and precisely estimated effect (SMD = 0.285, 95% CI: 0.200–0.369, *p* < 0.0001). Significant effects were also observed for RER (SMD = 0.263, 95% CI: 0.059–0.468, *p* = 0.0188) and VO_2_max (SMD = 0.386, 95% CI: 0.055–0.717, *p* = 0.0281). HR and MAP were borderline (*p* = 0.071 and *p* = 0.055, respectively), whereas 1RM, Peak Power, CMJ, and RPE were not statistically significant. Wide confidence intervals for CMJ and RPE indicate imprecision and limited evidence. Notably, when validated under the DerSimonian-Laird random-effects model, VO_2_max was the only outcome that remained relatively stable and statistically significant. Thus, BFR appears to yield a consistent improvement in VO_2_max, while effects on other outcomes should be interpreted cautiously.

#### 3.4.6 Comparative analysis of different BFR pressures

Meta-regression was performed after stratifying BFR pressure by measurement unit (mmHg vs. %AOP). Both linear and nonlinear (spline) analyses indicated no significant influence of BFR pressure on effect size (SMD). In linear models, neither the %AOP stratum (β = 0.0024, 95% CI: -0.0046–0.0095, *p* = 0.394) nor the mmHg stratum (β = −0.00134, 95% CI: −0.00405–0.00136, *p* = 0.286) showed a significant association, suggesting no stable linear dose–response relationship. Nonlinear patterns were explored using natural splines; the omnibus test was not significant in the mmHg stratum (*F* (3, 6) = 0.798, *p* = 0.538). Although one spline coefficient reached significance in the %AOP stratum, the limited number of independent clusters prevented a reliable omnibus Wald test; therefore, this apparent nonlinearity should be considered non-robust. Overall, current evidence does not support BFR pressure magnitude as a major moderator of BFR effects. Notably, this result may be statistically underpowered due to the limited sample size. Therefore, it should not be interpreted as evidence that BFR pressure exerts no moderating effect on physiological adaptations, but rather that the current data are insufficient to draw definitive conclusions.

#### 3.4.7 Robustness and dose–response consistency verification

To assess robustness, we performed supplementary analyses using the DerSimonian-Laird (DL) random-effects model alongside the primary three-level model with cluster-robust variance estimation (RVE). DL results were directionally consistent with the primary analysis and preserved the dose–response relationships between temporal variables and effect sizes, providing convergent support for the main inferences. The pooled DL effect was SMD = 0.231 (95% CI: 0.111–0.349), with no evidence of heterogeneity (*Q* = 37.861, *p* = 0.876), closely matching the RVE estimate in direction and magnitude. The primary difference was more conservative uncertainty estimation under the RVE framework. In intensity-stratified DL analyses, both high-intensity (SMD = 0.203) and low-intensity BFR (SMD = 0.272) showed small but statistically significant effects. The moderate-intensity subgroup had a larger point estimate but remained non-significant and heterogeneous (*k* = 9, *I*² ≈ 65.5%), indicating limited power and variability across studies. Temporal variables were also significant in DL models: BFR duration (β = 0.0133, 95% CI: 0.0009–0.0258, *p* = 0.0358) and stimulus duration (β = 0.0105, 95% CI: 0.0003–0.0207, *p* = 0.0446). Tertile-based subgroup analyses showed a positive gradient, consistent with dose–response trends from the primary RVE analysis. Outcome-stratified DL analyses indicated significant variability across outcomes (Q = 19.522, p = 0.021), with VO_2_max showing the most consistent improvement (SMD = 0.353). In contrast, other physiological outcomes did not reach statistical significance, suggesting that the effects on these parameters are tentative and lack robustness. Pressure-effect analyses (linear and spline), stratified by mmHg and %AOP, showed no clear dose–response pattern; given limited BFR pressure distributions and sample size constraints, this should be interpreted as insufficient evidence to detect a BFR pressure effect rather than evidence of no effect. Overall, while the DL sensitivity analyses supported the general directional trends of the primary RVE findings, the failure to fully reproduce statistical significance across multiple specific outcomes indicates that the overall findings are not universally robust. Instead, these comparative analyses confirm that only the improvements in VO_2_max demonstrate consistent statistical stability across different analytical models.

### 3.5 Publication bias analysis

#### 3.5.1 Low-intensity BFR group

The low-intensity BFR group included 7 independent studies contributing k = 15 effect sizes (**Figure 6**). Visual inspection of the funnel plot did not indicate marked asymmetry or clear one-sided missingness. Due to the limited number of independent trials and the dependence of the effect sizes, quantitative asymmetry testing was not performed; qualitative assessment did not reveal obvious small-study effects.

**Figure 6:**
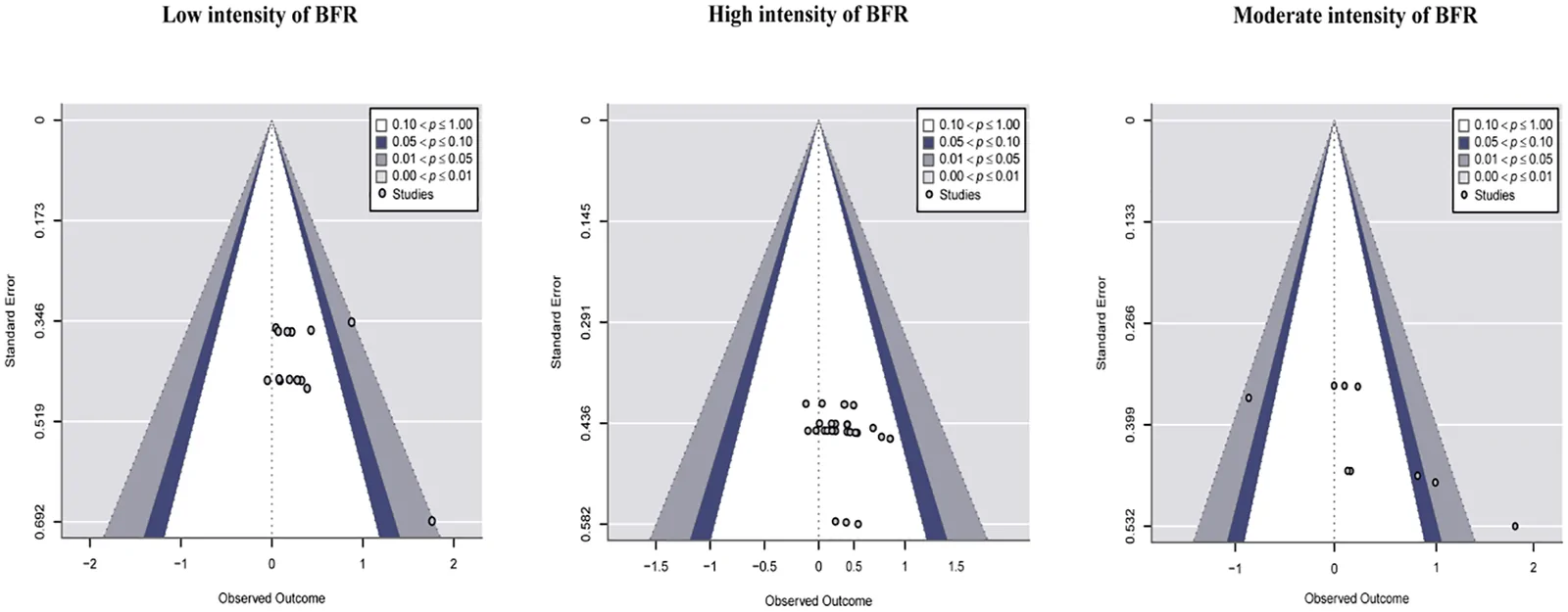
Funnel plots by BFR intensity.

#### 3.5.2 High-intensity BFR group

The high-intensity BFR group included 6 independent studies contributing k = 26 effect sizes. Similar to the low-intensity group, visual inspection of the funnel plot demonstrated a relatively symmetrical distribution without obvious clustering in statistically significant regions. The qualitative assessment did not indicate clear evidence of publication bias.

#### 3.5.3 Moderate-intensity BFR group

The moderate-intensity BFR group contained 3 independent studies contributing k = 9 effect sizes. Consistent with the other strata, we relied exclusively on a contour-enhanced funnel plot for qualitative assessment. Visual inspection did not suggest clear one-sided missingness, and the distribution did not show marked clustering in statistically significant regions. Given the extremely small evidence base and low precision, conclusions regarding publication bias for this group remain strictly exploratory.

### 3.6 Sensitivity analysis

A leave-one-out sensitivity analysis assessed the influence of individual studies on the pooled estimate. Across iterations, the pooled effect ranged from 0.2108 to 0.2955, indicating limited fluctuation and overall robustness. Except for one iteration (estimate = 0.2108, 95% CI: −0.0094–0.4311, p = 0.0606), the pooled effect remained statistically significant (*p* < 0.05) and consistently positive. This methodological sensitivity is largely attributable to the unique trial characteristics of Kakhak et al. (2022), which employed an outlier, prolonged BFR duration (55 min) and arbitrary, non-individualized cuff pressures rather than relative arterial occlusion pressures, thereby introducing protocol-driven heterogeneity. Across all iterations, τ² was estimated as 0, *I*² remained close to 0 (0–0.36%), and *Q*-tests were non-significant, suggesting minimal between-study heterogeneity and no single influential study. Notably, the removal of one specific study resulted in a loss of statistical significance (*p* = 0.0606). While the point estimate remained positive, demonstrating directional stability, it is crucial to distinguish this from the stability of statistical inference. This shift across the conventional significance threshold suggests that the overall statistical inference is somewhat fragile and reliant on specific trials, further warranting a cautious interpretation of the broad pooled effect.

## 4 Discussion

### 4.1 Dose–response relationship between hypoxic exposure duration and physiological adaptations

The stimulus elicited by BFR—including cumulative ischemic/hypoxic exposure, metabolite accumulation, and sustained afferent feedback—depends on the duration of different intervention phases. Appropriate control of BFR duration may intensify local anaerobic metabolism and acid–base disturbance, potentially facilitating recruitment of higher-threshold motor units even at relatively low external loads. Nevertheless, because available evidence is limited, conclusions about dose-dependent effects remain partly inconsistent. One 6-week longitudinal study reported no significant differences in maximal strength, fatigue resistance, or morphological outcomes (e.g., muscle thickness, cross-sectional area, or circumference) across different restriction durations, except for subjective pain perception (Schwiete et al., 2021). Furthermore, maintaining continuous occlusion during inter-set recovery periods has been suggested to impede full reperfusion and induce a ‘metabolic freeze,’ thereby sustaining intramuscular metabolic stress without requiring higher training intensities (Okita et al., 2019). In our univariate moderator analyses, BFR duration and stimulus duration were the only temporal variables reaching statistical significance. However, subsequent multivariable adjustments revealed that only total stimulus duration provides an independent physiological benefit. While we performed supplementary tertile-based subgroup analyses on the univariate data to visualize these initial patterns—which overall suggested a positive dose-response trend—these visual gradients likely reflect the aforementioned confounding effect rather than true independent physiological benefits. Mechanistically, longer inflation times are hypothesized to produce greater metabolic stress via repeated ischemia–reperfusion cycles, which represents a plausible mechanism for augmenting neuromuscular adaptation rather than a direct observation of the current meta-analysis. However, the non-significant effect in the medium-duration subgroup may reflect threshold behavior, uneven dose distribution, or limited statistical power. Similarly, tertile analyses of session duration suggested stronger effects in the medium- and long-duration groups, whereas the short-duration group did not reach significance. This may indicate that very short exposures provide insufficient additional metabolic stress to translate into measurable physiological gains. Variation in exercise modality, rest intervals, and intervention objectives across studies may also contribute to within-tertile heterogeneity. For example, Taylor et al. (Taylor et al, 2016) suggested that extending the intervention period from 2 to 4 weeks, alongside higher volume and longer sessions, may enhance outcomes, and Held et al. (Held et al, 2020) reported faster improvements in VO_2max_ with greater volume and longer sessions. In our univariate models, intervention period and frequency were not significant moderators. We present this lack of moderation as a finding specific to this review’s dataset, potentially because the ranges for these variables in the included literature were narrow and inconsistently reported. Importantly, indiscriminate application of BFR may increase risks such as hemodynamic disturbances and ischemia–reperfusion injury (Kakhak et al., 2022). Excessive hypoxia may also reduce hemoglobin and red blood cell counts, potentially contributing to anemia and impaired exercise capacity (Waclawovsky and Lehnen, 2016). Therefore, pressure, external load, and occlusion time should be carefully prescribed and monitored to avoid excessively low oxygen saturation and to ensure safety. It is important to note that the aforementioned physiological mechanisms, such as enhanced metabolic stress, repeated ischemia-reperfusion signaling, and greater motor-unit recruitment, are plausible hypotheses derived from broader exercise physiology literature. These pathways were not directly tested or quantified by the current meta-analysis and should be interpreted strictly as mechanistic possibilities rather than direct empirical proofs demonstrated by our findings.

### 4.2 Interaction between exercise intensity and adaptive responses

BFR can elicit adaptive improvements across a range of exercise intensities; however, the magnitude and consistency of benefits depend strongly on comparator conditions and analytical choices. Evidence suggests that low-intensity BFR can promote vascular and microvascular adaptations—characterized by greater lactate accumulation, enhanced skeletal muscle perfusion, and improved metabolite exchange—which may translate into improvements in selected physiological adaptations and physical performance (Amann et al., 2007; Larkin et al., 2012). By contrast, findings for high-intensity BFR are more variable. Some work suggests that higher intensity coupled with lower volume may suppress vascular endothelial growth factor (VEGF) expression and attenuate capillary formation (Gliemann et al., 2015), possibly because conventional high-intensity exercise already generates substantial metabolic stress and additional restriction may yield diminishing returns or increase fatigue. In this meta-analysis, BFR produced an overall small-to-moderate benefit (*k* = 50, *SMD* = 0.25). Stratified analyses indicated that low-intensity BFR conferred a clearer advantage over conventional low-intensity protocols; however, when compared with high-intensity exercise, adding blood flow restriction to low-intensity exercise did not consistently narrow physiological capability differences (Scott et al., 2015). High-intensity BFR was also statistically significant but with a smaller effect size, consistent with studies reporting similar improvements between high-intensity BFR and conventional high-intensity exercise (i.e., limited added benefit when intensity is already high) (Amani et al, 2018; Jukic et al., 2023). Moderate-intensity BFR showed a larger but non-significant point estimate with high heterogeneity, likely reflecting the small and diverse evidence base (three studies with varying controls, durations, and outcomes). Physiologically, BFR adaptations likely reflect an interaction of mechanical tension, metabolic stress, and neuromuscular activation; at moderate intensities, mismatched parameters may yield inconsistent responses. Overall, when explicitly considering comparator intensities, the findings support a pragmatic interpretation that low-intensity BFR provides the most robust added physiological benefit compared to conventional low-intensity exercise. In contrast, the additional marginal benefit of adding BFR to high-intensity exercise appears limited. Furthermore, generalized claims regarding the efficacy of ‘moderate-intensity BFR’ must be tempered, as the current evidence base for this specific stratum remains highly imprecise and its true effect uncertain.

### 4.3 Effects of BFR on exercise physiological adaptations

BFR imposes mechanical stimulation on the vascular wall, altering intraluminal tension and shear stress and modifying the microcirculatory milieu (e.g., reducing local oxygen partial pressure [PO_2_]), thereby influencing endothelial function (Loenneke et al., 2012). To maintain homeostasis, the body initiates neural, humoral, and local compensatory processes, during which physiological indicators and physical performance may also change (Pearson and Hussain, 2015). In our outcome-level analyses, periodic BFR was associated with improvements in some measures, suggesting potential benefits for physical performance. Outcome type explained part of the effect variation, with no significant residual heterogeneity (*Q*E(41) = 32.71, *p* = 0.819). Among outcomes, VO_2_max showed a stable and significant benefit (SMD = 0.386, 95% CI: 0.055–0.717, *p* = 0.0281), consistent with systematic reviews indicating that aerobic and intermittent BFR protocols can improve aerobic capacity (Formiga et al., 2020; Bennett and Slattery, 2019). Mechanistically, BFR may enhance peripheral oxygen utilization via local hypoxia and metabolic stress and induce vascular/microcirculatory adaptations; as a result, VO_2_max may be particularly sensitive to BFR stimuli. BFR under load may also promote skeletal muscle remodeling and increase capillary density through hypoxia-related signaling, while venous restriction–induced metabolite accumulation (e.g., lactate) can amplify metabolic stress, potentially improving aerobic endurance and aspects of anaerobic capacity (Ahmad et al., 2026; Gao et al., 2025; Yang et al., 2024; Li et al., 2024). Although MPO (SMD = 0.285, 95% CI: 0.200–0.369, *p* < 0.0001) and RER (SMD = 0.263, 95% CI: 0.059–0.468, *p* = 0.0188) were significant in the RVE framework, these effects were not statistically significant in the DL validation, suggesting sensitivity to sample size, the number of effect sizes, and protocol heterogeneity (e.g., intensity structure, task specificity, and neuromuscular stimulus) or potential ceiling effects in highly trained participants. Unlike VO_2_max, purely strength- and power-related outcomes and neuromuscular variables such as CMJ, Peak Power, and 1RM depend more strongly on stimuli such as high mechanical tension, stretch–shortening cycle loading, and neural drive. If BFR protocols are embedded primarily within endurance or interval frameworks, or if movement patterns and intensities are not aligned with power-based assessments, physiological adaptations may not translate into consistent improvements in these neuromuscular metrics. This interpretation aligns with prior evidence that BFR effects on strength and hypertrophy are context-dependent and influenced by loading parameters and participant characteristics (Lixandrão et al., 2018; Patterson et al., 2019). For 1RM, Peak Power, MAP, and RPE, confidence intervals crossed zero, indicating insufficient evidence to exclude null or small effects. Moreover, measures such as MAP, RER, and RPE can be influenced by acute testing context and timing; transient cardiovascular or hemodynamic stress during BFR does not necessarily imply long-term adaptation. Heterogeneity in BFR pressure, occlusion duration, exercise modality, and participant characteristics likely further contributes to uncertainty in these estimates (Hughes et al., 2017; Lixandrão et al., 2018; Stølen et al., 2005). Thus, VO_2_max is the only outcome demonstrating a relatively robust improvement in the current evidence base, while effects on other outcomes appear more protocol- and context-dependent. Quantitatively, our pooled VO_2_max estimate (SMD = 0.386) aligns with Gao et al. (2025) (aerobic capacity SMD = 0.27), yet remains notably more conservative than the exceptionally large effects reported by Yang et al. (2024) (endurance ES = 1.39–1.40) and Li et al. (2024) (VO_2_max SMD = 1.65). This modest magnitude likely reflects our stricter inclusion criteria (exclusively active cohorts) and rigorous multilevel adjustments for dependent effect sizes. Crucially, we extend this evidence base by establishing that these aerobic adaptations are profoundly dose-dependent on accumulated stimulus duration.

### 4.4 Limitations

Several limitations regarding the evidence base, analytical framework, and study designs warrant careful consideration. First, the current evidence base is relatively small (16 independent trials yielding 50 effect sizes), resulting in sparse moderator strata that inherently constrain statistical power during subgroup analyses. Second, our meta-regression analyses are observational by nature; therefore, the identified dose-response relationships cannot establish direct causality, and the duration metrics must be regarded strictly as indirect moderating variables rather than direct causal drivers. While we employed multivariable meta-regression to address the observed collinearity between session-level durations and the total intervention period, the primary univariate associations remain susceptible to confounding from underlying protocol variables such as exercise modality, training volume, and rest intervals. Third, to maintain macroscopic analytical feasibility, we did not apply statistical corrections for multiplicity (multiple testing), which theoretically inflates the risk of Type I errors across our numerous moderator and outcome assessments. Fourth, pooling diverse cardiopulmonary and neuromuscular outcomes into a single global index of ‘physiological adaptation’ was necessary for temporal dose-response modeling, but this operational definition inevitably obscures the mechanistic specificity of distinct biological pathways. Furthermore, demographic and methodological constraints limit the generalizability of our findings. No included study exclusively investigated female cohorts, precluding sex-specific inferences, and participant age was structurally bounded to a minimum of 15.9 years (Kakhak et al., 2022). Consequently, the present findings are applicable solely to physically active adolescents and athletes, and cannot be extrapolated to older adults, clinical rehabilitation populations, or exclusively female cohorts. This highlights a critical gap, as dose–response temporal questions remain open and clinically relevant in these neurological and clinical populations, motivating future reviews (Feng et al., 2025). The relatively short intervention periods (averaging 4.5 weeks) restrict conclusions to short-term adaptations. Crucially, none of the primary studies systematically reported adverse-event data, rendering a quantitative safety analysis of these ischemic protocols impossible. Finally, restricting our search to English-language publications may have introduced language bias. Additionally, the omission of gray literature and unpublished trial registries (e.g., ClinicalTrials.gov) introduces potential dissemination bias, which may lead to an overestimation of the intervention effects. Relying on a single reviewer for the initial title/abstract screening introduced a theoretical risk of study omission, despite rigorous dual-reviewer verification in all subsequent stages.

## 5 Conclusions

Several time-related variables in BFR appear to influence improvements in exercise physiological capacities. Our results suggest positive dose–response relationships between effect size and both BFR duration and stimulus duration. However, multivariable models revealed that the apparent benefit of prolonged BFR duration is largely confounded by the total intervention period, cautioning against indiscriminately extending ischemic exposure per session. Other potential moderators (exposure frequency, intervention period, and BFR pressure) were not statistically significant. Regarding intensity, low-intensity BFR showed a clearer advantage over conventional low-intensity exercise, whereas high-intensity BFR provided relatively smaller additional benefits compared with conventional high-intensity protocols. Moderate-intensity BFR showed a potentially beneficial point estimate but did not reach statistical significance. Across outcomes, VO_2_max, MPO, and RER were significant in the RVE framework, HR and MAP were borderline, and remaining outcomes were non-significant; validation analyses indicated that only VO_2_max showed a relatively stable improvement. Therefore, broad claims implying generally robust physiological adaptations should be avoided. Instead, we emphasize that the strongest and most consistent evidence within the current literature relates specifically to improvements in VO_2_max. Consistency checks using the DerSimonian-Laird random-effects model supported the robustness of these inferences. Dose–response analyses suggested larger effects with longer stimulus duration and BFR duration within the included evidence base; however, the apparent thresholds (stimulus duration > 30 min and BFR duration > 19.5 min) were derived from data-driven tertile splits. Therefore, they must remain solely as hypothesis-generating observations, and should not be interpreted as physiologically validated cut-points until rigorously confirmed in prospective trials. This may elicit greater physiological stimulation under relatively low external load while supporting physiological efficiency; however, it must be emphasized that the safety of these protocols could not be systematically evaluated due to the lack of adverse-event reporting in the primary literature. Future studies should increase sample sizes, optimize study design, and improve outcome reporting to clarify longer-term adaptations and parameter-specific effects. Furthermore, future syntheses should examine whether temporal dose–response patterns extend to inactive and clinical populations (Ahmad et al., 2026; Feng et al., 2025).
