# Supplementary material for: Dose–response relationships between temporal variables of blood flow restriction exercise and physiological adaptations: a systematic review and meta-analysis
Source: Front Physiol. 2026 Oct 1;17:1947412. doi: 10.3389/fphys.2026.1947412 (PMC13669561; doi:10.3389/fphys.2026.1947412)
Supplement: Supplementary file 1 [file DataSheet1.docx]

**Supplementary Table S1: Database-specific Search Strategies**

**1. PubMed (NLM)**

Search syntax tailored for Title/Abstract and MeSH terms.

#1 Intervention: "Blood Flow Restriction"[Title/Abstract] OR "Blood Flow Restricted"[Title/Abstract] OR "BFR"[Title/Abstract] OR "BFRT"[Title/Abstract] OR "KAATSU"[Title/Abstract] OR "Vascular Occlusion"[Title/Abstract] OR "Ischemic Training"[Title/Abstract]

#2 Outcomes: "Oxygen Consumption"[MeSH Terms] OR "VO2max"[Title/Abstract] OR "Cardiorespiratory Fitness"[Title/Abstract] OR "Muscle Strength"[MeSH Terms] OR "Strength"[Title/Abstract] OR "1RM"[Title/Abstract] OR "Peak Power"[Title/Abstract] OR "Countermovement Jump"[Title/Abstract] OR "CMJ"[Title/Abstract] OR "Heart Rate"[MeSH Terms] OR "Respiratory Exchange Ratio"[Title/Abstract] OR "RER"[Title/Abstract] OR "Physical Exertion"[MeSH Terms] OR "RPE"[Title/Abstract] OR "Athletic Performance"[MeSH Terms] OR "Sport Performance"[Title/Abstract] OR "Exercise Capacity"[Title/Abstract]

#3 Final Search: #1 AND #2 (Filters: English)

**2. Web of Science (Core Collection)**

Search syntax using Topic (TS) which searches Title, Abstract, Author Keywords, and Keywords Plus.

#1 Intervention: TS=("Blood Flow Restriction" OR "Blood Flow Restricted" OR "BFR" OR "BFRT" OR "KAATSU" OR "Vascular Occlusion" OR "Ischemic Training")

#2 Outcomes: TS=("VO2max" OR "Oxygen Consumption" OR "Cardiorespiratory Fitness" OR "Muscle Strength" OR "1RM" OR "Peak Power" OR "Countermovement Jump" OR "CMJ" OR "Heart Rate" OR "Respiratory Exchange Ratio" OR "RER" OR "Perceived Exertion" OR "RPE" OR "Athletic Performance" OR "Sport Performance" OR "Exercise Capacity")

#3 Final Search: #1 AND #2 (Refined by: Languages=ENGLISH)

**3. Scopus (Elsevier)**

Search syntax using TITLE-ABS-KEY.

#1 Intervention: TITLE-ABS-KEY ("Blood Flow Restriction" OR "Blood Flow Restricted" OR "BFR" OR "BFRT" OR "KAATSU" OR "Vascular Occlusion" OR "Ischemic Training")

#2 Outcomes: TITLE-ABS-KEY ("VO2max" OR "Oxygen Consumption" OR "Cardiorespiratory Fitness" OR "Muscle Strength" OR "1RM" OR "Peak Power" OR "Countermovement Jump" OR "CMJ" OR "Heart Rate" OR "Respiratory Exchange Ratio" OR "RER" OR "Perceived Exertion" OR "RPE" OR "Athletic Performance" OR "Sport Performance" OR "Exercise Capacity")

#3 Final Search: #1 AND #2 AND LIMIT-TO (LANGUAGE, "English")

**4. SPORTDiscus (EBSCOhost)**

Search syntax using SU (Subject Terms) and TI/AB (Title/Abstract).

#1 Intervention: TI ("Blood Flow Restriction" OR "Blood Flow Restricted" OR "BFR" OR "BFRT" OR "KAATSU" OR "Vascular Occlusion") OR AB ("Blood Flow Restriction" OR "Blood Flow Restricted" OR "BFR" OR "BFRT" OR "KAATSU" OR "Vascular Occlusion")

#2 Outcomes: TI ("VO2max" OR "Oxygen Consumption" OR "Muscle Strength" OR "1RM" OR "Peak Power" OR "CMJ" OR "Heart Rate" OR "RER" OR "RPE" OR "Athletic Performance" OR "Exercise Capacity") OR AB ("VO2max" OR "Oxygen Consumption" OR "Muscle Strength" OR "1RM" OR "Peak Power" OR "CMJ" OR "Heart Rate" OR "RER" OR "RPE" OR "Athletic Performance" OR "Exercise Capacity")

#3 Final Search: #1 AND #2 (Limiters - Language: English)
